# Advances in Forensic Toxicology in Veterinary Medicine: Current Perspectives, Analytical Progress, and Future Challenges

**DOI:** 10.3390/vetsci13050444

**Published:** 2026-04-30

**Authors:** Giulio Mannocchi, Filippo Roberto Busardò, Luigi Tonino Marsella, Roberta Tittarelli

**Affiliations:** 1 Department of Biomedicine and Prevention, Section of Legal Medicine, Social Security and Forensic Toxicology, Faculty of Medicine and Surgery, University of Rome “Tor Vergata”, Via Montpellier 1, 00133 Rome, Italy; marsella@uniroma2.it (L.T.M.); roberta.tittarelli@uniroma2.it (R.T.); 2 Veterinary Diagnostics North Rome, Via Roccaraso 7/c, 00135 Rome, Italy; filippo.busardo@hotmail.it

**Keywords:** veterinary forensic toxicology, animal poisoning, wildlife poisoning, forensic veterinary medicine, one health, mass spectrometry

## Abstract

Over the past decade, veterinary forensic toxicology has gained increasing relevance due to the recognition of animal poisoning as a criminal, environmental, and public health concern. This review provides an updated overview on current perspectives in veterinary forensic toxicology, focusing on common toxicological scenarios, analytical innovations, and interpretative challenges. Intentional poisoning of companion animals, wildlife intoxication, and environmental contamination are discussed within a One Health framework. Mass spectrometry-based techniques, including targeted gas chromatography–tandem mass spectrometry (GC-MS/MS), liquid chromatography–tandem mass spectrometry (LC–MS/MS) and high-resolution mass spectrometry (HRMS) for non-targeted screening, and emerging ambient mass spectrometry approaches are highlighted as pivotal tools in modern forensic investigations. Major limitations related to post-mortem changes, species-specific toxicokinetic, and the lack of harmonized interpretative criteria are critically examined. The review also identified future priorities such as methodological standardization, expansion of toxicokinetic databases across species, detection of emerging contaminants, and strengthened international cooperation. Overall, veterinary forensic toxicology is an emerging discipline at the intersection of animal welfare, environmental conservation, and public health providing essential evidence for legal, regulatory, and investigative purposes.

## Simple Summary

Veterinary forensic toxicology is an interdisciplinary field that supports the investigation of suspected animal poisoning in legal, regulatory and environmental contexts. This review provides an updated overview of recent developments in the discipline, emphasizing the most common poisoning scenarios involving companion animals and wildlife. Particular attention is given to advances in analytical strategies currently applied in forensic casework based on gas chromatography–tandem mass spectrometry (GC-MS/MS), liquid chromatography–tandem mass spectrometry (LC–MS/MS) and high-resolution (HRMS) platforms, which have significantly improved the detection of both known and unexpected toxicants in post-mortem samples. Despite these technological improvements, significant challenges remain in the interpretation of toxicological results, including post-mortem degradation, limited species-specific reference data, and the absence of standardized cut-off values. From a One Health perspective, veterinary forensic toxicology plays a crucial role in detecting illegal practices, protecting biodiversity, and preventing risks to human health. Continued methodological innovation, harmonization of protocols, and international collaboration are essential to ensure the reliability and forensic relevance of toxicological evidence in veterinary medicine.

## 1. Introduction

Veterinary forensic toxicology is an interdisciplinary discipline integrating veterinary pathology, analytical chemistry, environmental sciences, and legal medicine. It applies toxicological principles and methodologies to investigations involving animals within legal, regulatory, and medico-legal contexts [1]. Its importance has grown substantially due to the persistent occurrence of intentional poisonings in companion animals [2,3,4,5,6,7,8,9,10,11], large-scale poisoning events affecting wildlife and livestock [10,11,12,13,14,15,16,17,18,19,20,21,22], and emerging scenarios involving pharmaceuticals and drugs of abuse [15,17,23,24].

Animals function as effective sentinels of environmental contamination and illegal chemical use, providing valuable information for environmental risk assessment and public health protection [16,17,20]. Within the One Health framework, veterinary forensic toxicology links animal, environmental, and human health, enabling the detection of chemical hazards that may affect multiple species simultaneously.

In forensic contexts, toxicology extends beyond its traditional clinical role and acquires an evidentiary dimension. The aim is not only to detect and identify toxic substances but also to reconstruct exposure scenarios, assess causality in injury or death, and evaluate intent, negligence, or regulatory non-compliance [25]. Toxicological findings therefore frequently inform judicial, regulatory, and administrative decision-making.

Over the last decade, veterinary forensic toxicology has undergone significant conceptual and methodological development. Dedicated reviews, methodological studies on post-mortem toxicology, and thematic issues addressing animal poisoning and biomarkers of exposure have contributed to its formal recognition and consolidation [2,3,5,13,14,15,16,24,26,27,28,29]. This discipline has progressively expanded beyond its historically established hubs in the United States and the United Kingdom, with increasing contributions from South America, Europe, Asia, and the Caribbean, reflecting its growing global recognition and legal and societal relevance [30]. Advances in wildlife toxicology, particularly in Europe, have documented the impacts of pesticides, veterinary pharmaceuticals, and toxic metals on free-ranging species, emphasizing the roles of illegal poisoned baits, secondary exposure, and chronic or sub-lethal intoxication [10,11,17,24,31,32,33,34]. The establishment of coordinated European networks of analytical laboratories and governmental agencies has further demonstrated the integration of forensic toxicology into biodiversity conservation and regulatory enforcement strategies [16].

The scope of veterinary forensic toxicology extends beyond overt criminal poisoning. Toxicological evidence is increasingly relevant in civil disputes, livestock production oversight, and wildlife protection, where it supports legal actions under environmental and conservation legislation [35]. Veterinary forensic medicine, encompassing disciplines as anatomy, toxicology, pathology, genetics, and forensic entomology has gained formal recognition in several countries, although structural and organizational limitations still persist [16,36]. The integration of veterinary medicine with investigative procedures, analytical chemistry, ethical considerations and legal frameworks constitutes the foundation for accurately determining the causes and the circumstances of an animal’s death [7].

A significant milestone in this field has been the publication of the first consensus guideline for veterinary forensic post-mortem examinations (ANSI/ASB Standard 170, First Edition published in 2024). This document establishes a standardized framework for forensic procedures, thereby enhancing the scientific rigor and legal robustness of veterinary forensic investigations [37].

This review provides an updated overview of advances in veterinary forensic toxicology from 2015 to 2025. The current work provides addresses for post-mortem investigations, recent analytical developments based on advanced instrumentation including LC-DAD, [3,26], GC/MS [18,38], GC-MS/MS [18,39,40], LC-MS [41], LC-MS/MS [18,27,40,42,43,44,45], LC-QTOF [17,22,27,28,46], DART-HRMS [12,13], as well as the main classes of toxicants encountered in practice. Furthermore, it discusses the key interpretative and organizational challenges that must be addressed to ensure the development and the legal recognition of the discipline.

## 2. Methodology

A comprehensive literature search was performed using major electronic databases, including PubMed, Scopus and Web of Science, covering the period from January 2015 to December 2025. The search strategy was designed to identify relevant publications in the field of veterinary forensic toxicology by combining controlled vocabulary terms and free-text keywords.

The following keywords were used: “veterinary forensic toxicology”, “animal poisoning”, “wildlife poisoning”, and “forensic veterinary medicine”. These terms were combined using Boolean operators. In particular, OR was used to include synonymous or related terms (e.g., “animal poisoning” OR “wildlife poisoning”), while AND was applied to combine different conceptual domains (e.g., “veterinary forensic toxicology” AND “animal poisoning”). Additional search strings included (“veterinary forensic toxicology” AND “analytical methods”), (“wildlife poisoning” AND “forensic analysis”), and (“animal poisoning” AND (“GC-MS” OR “LC-MS/MS” OR “HRMS”).

The search was limited to peer-reviewed articles published in English. Both original research articles and review papers were considered. The reference lists of selected articles were also manually screened to identify additional relevant studies.

Inclusion criteria comprised studies focusing on veterinary forensic toxicology, animal and wildlife poisoning, analytical methodologies, and forensic applications in veterinary medicine. Exclusion criteria included studies not directly relevant to the topic, articles lacking sufficient methodological detail, conference abstracts, and publications not available in English.

## 3. Results

### 3.1. Forensic Toxicology of Poisoning in Companion Animals and Wildlife

Dogs and cats are the species most frequently involved in veterinary forensic investigations for suspected poisoning, reflecting their close proximity to human environments and their high likelihood of both accidental and intentional exposure. Carbamate pesticides, including carbofuran and aldicarb, remain the most commonly detected toxic agents, despite regulatory bans [2,3,26]. These compounds exert their toxicity primarily through reversible inhibition of acetyl-cholinesterase (AChE), leading to accumulation of acetylcholine at synaptic junctions and subsequent overstimulation of muscarinic and nicotinic receptors. This leads to a cholinergic toxidrome characterized by hypersalivation, tremors, bronchoconstriction, seizures, and potentially fatal respiratory failure. Their high acute toxicity, rapid onset of action, and frequent use in illegally prepared baits contribute to their forensic relevance [47].

Other frequently encountered toxicants include molluscicides such as metaldehyde [4], metallic phosphides [5,39], and pyrethroid insecticides [29,36]. These agents may induce severe or fatal intoxication through mechanisms involving neurotoxicity, disruption of cellular respiration, or oxidative stress mediated by disturbances in neurotransmitter balance, particularly reduced γ-aminobutyric acid (GABA) activity, leading to uncontrolled neuronal excitation, tremors, hyperthermia, and metabolic acidosis. Although pyrethroids are generally less toxic to mammals, they can cause significant neurotoxic effects in companion animals due to limited hepatic glucuronidation, acting by prolonging the opening of voltage-gated sodium channels, resulting in repetitive neuronal firing, tremors, and seizures [48].

A critical limitation affecting these classes of toxicants emerging across studies is the lack of standardized toxicological thresholds and species-specific reference values. This significantly constrains the interpretation of toxicological results, particularly in distinguishing between lethal, sub-lethal, and incidental exposure. In wildlife investigations, this issue is further compounded by ecological variability and the frequent occurrence of secondary poisoning, which complicates causal attribution.

In addition to these agents, several household toxicants are increasingly reported in companion animal poisoning cases, including xylitol [49], grapes and raisins [50], chocolate [51], lilies [52], and ethylene glycol [53]. These substances are readily accessible in domestic environments and may cause severe clinical outcomes, ranging from acute renal failure (grapes/raisins, lilies, ethylene glycol) to hypoglycaemia and hepatic failure (xylitol), and cardiotoxic or neurologic effects (chocolate).

In recent years, exposure to pharmaceuticals and drugs of abuse has become an increasing concern in veterinary forensic toxicology. Documented cases involving amphetamines, methamphetamines, cannabis, and synthetic cannabinoids in dogs and cats underscore the evolving nature of poisoning scenarios.

Amphetamines and methamphetamines increase synaptic concentrations of monoamines (dopamine, norepinephrine, and serotonin), producing sympathomimetic effects such as tachycardia, hyperthermia, agitation, and seizures. Cannabis intoxication, primarily mediated via CB_1_ receptor agonism, typically presents with central nervous system depression, ataxia, bradycardia, and urinary incontinence, although paradoxical excitation may occur. Synthetic cannabinoids, often more potent and unpredictable, are associated with severe neurotoxicity, including seizures and coma. These trends highlight the necessity of advanced analytical strategies, including LC-MS/MS and high-resolution mass spectrometry (HRMS), to enable both targeted and untargeted detection of a wide spectrum of xenobiotics in biological matrices such as blood, urine, liver, and gastric contents [54].

The increasing complexity of poisoning cases highlights the importance of advanced analytical approaches that can detect unconventional or emerging toxicants in veterinary contexts, reflecting changes in human behaviour and substance availability [15,23,24].

A related but distinct concern is wildlife poisoning, which poses significant challenges for both conservation biology and forensic investigations. Birds of prey and scavengers are particularly vulnerable due to their ecological roles and feeding behaviour. Anticoagulant rodenticides (ARs) are widely detected in raptors and are responsible for both lethal and sub-lethal effects [9,11,14,20,21,22,40,43,44,45,46,55,56].

These compounds inhibit vitamin K epoxide reductase (VKORC1), impairing the recycling of vitamin K and preventing the activation of clotting factors II, VII, IX, and X. The resulting coagulopathy manifests as spontaneous hemorrhages and heightened susceptibility to trauma. Second-generation ARs, characterized by high lipophilicity, long biological half-lives, and significant hepatic accumulation, increase the risk of secondary poisoning in predatory and scavenging species.

Beyond ARs, a broad spectrum of other toxicants have been reported in wildlife cases, including strychnine [11], carbamates, organophosphates, neonicotinoids, pyrethroids, and banned pesticides [2,3,10,27,29,36]. These compounds often reflect environmental contamination, illegal poisoning campaigns, or secondary exposure through trophic transfer. Strychnine acts as a competitive antagonist of glycine receptors in the spinal cord and brainstem, resulting in unchecked excitatory neurotransmission and severe tonic convulsions. Organophosphates share a similar mechanism with carbamates but produce irreversible AChE inhibition, often leading to prolonged toxicity. Neonicotinoids, acting as nicotinic acetylcholine receptor agonists, are particularly toxic to insects but may also affect non-target vertebrates through chronic exposure. Heavy metals such as lead remain a persistent problem in scavenging species due to ammunition ingestion. Lead toxicosis is well documented to cause neurological impairment, gastrointestinal dysfunction, and mortality, with significant implications at the population level [55,57].

Additionally, veterinary pharmaceuticals, including non-steroidal anti-inflammatory drugs (NSAIDs) and antibiotics, have been increasingly detected in avian scavengers, raising concerns about chronic exposure and population-level effects [17,28].

Beyond these well-documented contaminants, veterinary sedatives represent an emerging concern in wildlife forensic investigations. In particular, medetomidine, a chiral α_2_-adrenergic agonist, is gaining attention due to its use in immobilization procedures and its potential misuse or unintended environmental exposure [41]. The presence of these agents highlights the increasing complexity of forensic toxicology in veterinary contexts, where both intentional and indirect exposures must be considered within a multidisciplinary investigative approach. Table 1 provides detailed information about the main classes of substances involved in veterinary forensic toxicological cases.

Overall, the convergence of traditional toxicants, emerging contaminants, and anthropogenic influences highlights the need for continuous methodological advancement and enhanced surveillance in veterinary forensic toxicology [58]. This requires not only the improvement of analytical methodologies but also a more effective integration of toxicological findings, pathological data, and environmental context to accurately reconstruct poisoning events in both companion animals and wildlife.

In addition, emerging environmental contaminants are increasingly recognized in wildlife forensic toxicology. So-called “forever chemicals,” particularly per- and polyfluoroalkyl substances (PFAS), are characterized by extreme environmental persistence and bioaccumulation, and have been widely detected in aquatic and terrestrial wildlife, where they may induce immunotoxic, endocrine, and developmental effects [59]. Harmful algal blooms, especially those involving cyanobacteria, represent another significant source of intoxication, producing cyanotoxins such as microcystins and anatoxins, which can cause acute hepatotoxicity and neurotoxicity in domestic and wild animals following ingestion of contaminated water [60]. Furthermore, mercury remains a critical toxicant in wildlife, particularly in aquatic ecosystems, where methylmercury bioaccumulates along trophic chains, leading to severe neurological impairment, reproductive failure, and population-level effects in predatory species [61].

In addition, while the growing detection of pharmaceuticals and drugs of abuse reflects evolving exposure scenarios, the literature remains fragmented, with limited systematic investigations and scarce toxicokinetic data. This represents a major gap in understanding the real forensic relevance of these emerging contaminants.

### 3.2. Analytical Progress in Veterinary Forensic Toxicology

Recent analytical developments in veterinary forensic toxicology have been largely associated with the implementation of mass spectrometric techniques, enabling both targeted and untargeted detection of a wide spectrum of toxicants in complex biological matrices.

While the literature extensively describes the application of these methodologies, a comparative evaluation highlights important differences in performance, applicability, and limitations across analytical platforms.

Gas chromatography coupled to mass spectrometry (GC-MS and GC-MS/MS) remains a fundamental analytical approach in post-mortem investigations, particularly for the identification of volatile and thermally stable compounds such as pesticides, rodenticides, fumigants, and related environmental contaminants. Its main strengths include high reproducibility, relatively low operational costs, and the availability of extensive spectral libraries.

In a retrospective analysis of animal poisoning cases from 2009 to 2018 Avolio et al., developed and validated a GC-MS method for the detection and quantification of volatile compounds including organochlorines (α-endosulfan, β-endosulfan, endosulfan-sulfate and lindane) and pyrethroids (cypermethrin, deltamethrin, permethrin I and II) [25]. The method was also validated for the determination of ethylene glycol, piperonyl butoxide and embutramide. The species most frequently involved in suspected poisoning cases, in decreasing order, were dogs, cats, synanthropic birds such as mallards and pigeons, livestock (including bees, donkeys, cattle, goats, rabbits, horses, sheep, chickens and pigs) and wildlife.

Focardi et al. [18] described a comprehensive Systematic Toxicological Analysis (STA) approach in which GC-MS was used for untargeted screening of toxicants in baits and biological matrices and LC-MS/MS for confirmatory analysis.

GC-MS, supported by deconvolution software (AMDIS version 2.73), enabled wide-spectrum detection, confirming its suitability as a primary screening technique in complex forensic samples.

However, the limitations of conventional GC–MS in terms of selectivity, especially for highly volatile compounds, are highlighted by Lehner et al. [39], who developed a headspace GC–MS/MS method for phosphine detection. In this case, tandem mass spectrometry with collision-induced dissociation allowed the identification of specific ion transitions (e.g., *m*/*z* 34 → 31, 32, 33) and the optimization of multiple reaction monitoring (MRM) conditions, significantly improving analytical specificity and reducing interference from air components. The method demonstrated adequate sensitivity and reliability over a working range of 5–100 ppm, without requiring chromatographic separation from atmospheric gases, thus representing a clear advancement over single-stage GC–MS for confirmatory analysis of gaseous toxicants.

Rial-Berriel et al. [40] further extended the applicability of gas chromatographic–mass spectrometric techniques by developing a method for the simultaneous determination of 351 contaminants, of which 126 were analysed using GC-MS/MS and 245 substances were analysed using LC-MS/MS. In this context, GC–MS contributes to the analysis of persistent organic pollutants and semi-volatile compounds within a comprehensive screening strategy characterized by very low limits of quantification (80% < 2 ng/g). Altogether, these studies demonstrate that while GC–MS remains fundamental for wide-scope screening due to its versatility and compatibility with complex matrices, GC–MS/MS—particularly in headspace and MRM mode—provides superior selectivity and sensitivity for specific analytes, supporting a complementary and integrated instrumental approach in veterinary forensic toxicology and biomonitoring.

These analytical techniques are frequently combined with QuEChERS-based extraction protocols to improve analyte recovery and matrix purification, thereby enhancing analytical performance in terms of sensitivity, selectivity, and reproducibility [18,38,39,40,55,62]. The increasing complexity and variability of poisoning scenarios have led to the extensive application of liquid chromatography-based methodologies. Liquid chromatography–tandem mass spectrometry (LC-MS/MS) has become the reference technique for targeted multi-residue analysis, due to its high sensitivity, selectivity, and robustness in the quantification of multiple classes of compounds, including anticoagulant rodenticides, veterinary pharmaceuticals, mycotoxins, and pesticides, even at trace concentrations in complex biological samples [18,42,43,44,45,63].

Avolio et al. performed forensic toxicological analyses on 851 samples, including 497 collected from suspected animal poisoning cases and 354 from suspected poisoned baits. The authors applied a modified QuEChERS approach to streamline sample preparation, followed by LC-MS analyses for the detection of various classes of toxicants, including carbamates, anticoagulant rodenticides, strychnine, α-chloralose. The compounds most frequently involved in intentional poisoning cases were anticoagulant rodenticides—such as coumatetralyl, brodifacoum, bromadiolone, and difenacoum—non-anticoagulant rodenticides (e.g., α-chloralose), and molluscicides, particularly metaldehyde [25]. Zhu et al. also employed a QuEChERS-based approach for the extraction and purification of anticoagulant rodenticides from animal tissues, including sheep whole blood, heart, liver, kidney, muscle, and stomach wall. The analytical determination was performed using high-performance liquid chromatography coupled with triple quadrupole/linear ion trap tandem mass spectrometry (HPLC-QTrap-MS/MS). Thirteen rodenticides (coumafuryl, valone, pindone, coumatetralyl, warfarin, coumachlor, diphacinone, dicoumarol, chlorophacinone, bromadiolone, difenacoum, flocoumafen, and brodifacoum) were analyzed using a fully validated method demonstrating high selectivity, precision, and accuracy, with linearity over the range of 1–100 ng/mL (g), while minimal matrix effects (≤20%) were observed for the majority of analytes. This approach enhanced analytical sensitivity and selectivity, improving the detection and confirmation of anticoagulant rodenticides in complex biological matrices representing a valuable tool for the investigation of rodenticide poisoning cases in forensic contexts [64].

Taylor et al. validated an analytical strategy that expanded the screened panel to 159 multi-class compounds, including pesticides, veterinary drugs, and anticoagulant rodenticides, in tissues from non-target vertebrate animals, with linearity in the range 0.0005–0.05 μg/mL. The method demonstrated robust selectivity, precision, and accuracy across different matrices (liver, muscle, and kidney) with satisfactory recoveries, making it particularly suitable for large-scale monitoring of hundreds of suspected poisoning incidents [43].

Building on these developments, Drague et al. reported the validation of a sensitive LC-MS/MS assay capable of simultaneously quantifying eighteen therapeutic oral anticoagulants, rodenticides, and antiplatelet agents. The method required minimal sample volume and employed a simple pre-treatment protocol. Its applicability to different biological matrices—including whole blood, plasma, bile, and vitreous humor—ensured reliable quantification across complex specimens, providing robust support for forensic, clinical, and toxicological investigations [65].

Recently, Fernandez-Garcìa et al., developed and validated a new analytical method based on LC-MS/MS for the detection and quantification of 112 organic compounds, including pesticides, anticoagulant rodenticides, and per- and polyfluoroalkyl substances (PFASs), in the blood of four top-predator birds. They used a miniaturized liquid–liquid extraction, determining the presence of these compounds at trace concentration levels using a low volume of blood (200 μL) and ensuring non-destructive sampling [66].

Moreover, liquid chromatography coupled with high-resolution mass spectrometry (LC-HRMS), including time-of-flight (TOF) and Orbitrap analyzers, provides accurate mass measurements, improving confidence in compound identification. These approaches support advanced analytical strategies by using spectral libraries and retrospective interrogation of acquired data for the detection of unknown, unexpected, or emerging toxicants in both post-mortem and in vivo samples [17,22,27,28].

Gao et al. reported a novel methodology for the determination of nine anticoagulant rodenticides (brodifacoum, bromadiolone, warfarin, coumachlor, coumatetralyl, difenacoum, pindone, difethialone and flocoumafen) in blood, combining supported liquid extraction (SLE) pre-treatment with LC-HRMS/MS analysis in parallel reaction monitoring (PRM) on a Q-Orbitrap platform. This approach enabled the quantification of these compounds at trace concentrations ranging from 0.006 to 0.002 ng/mL. This method allowed the detection of minimal residues of these toxic agents, making it applicable for both veterinary and human toxicology applications [67].

Furthermore, innovative ambient ionization techniques such as direct analysis in real time coupled with high-resolution mass spectrometry (DART-HRMS) have introduced rapid screening methodologies that require minimal sample preparation. This innovative approach significantly reduces the time of analysis maintaining the ability to generate informative chemical profiles, representing a valuable tool in time sensitive forensic investigations. [12,13]. Nevertheless, their use remains largely exploratory, and further validation is required to establish their reliability, reproducibility, and forensic admissibility.

A DART-HRMS approach was used by Zacometti et al. for the detection of toxic alkaloids in animal autopsy specimens. The method was successfully applied to rumen contents, liver specimens, visible seeds and leaves retrieved from the autopsy, revealing the presence of Calycanthaceae alkaloids, in particular calycanthine, folicanthidine, and calycanthidine.

HPLC-HRMS/MS was used to confirm the presence of calycanthine in both rumen contents and liver specimens and enabled its quantification, with concentrations in liver ranging from 21.3 to 46.9 mg·kg^−1^. This study represents the first report of calycanthine quantification in liver following a fatal intoxication event [12].

Pozzato et al. also reported a significant forensic application of DART-HRMS by identifying mycotoxin asperphenamate, produced by fungi of the genera Penicillium and Aspergillus, in a batch of hay consumed by cattle.

Yu et al. applied DART-ToF for ultra-rapid screening of water-soluble anticoagulant rodenticides in environmental samples, using an in-house spectral library and ionization mode selection (positive or negative) for each compound. This approach enabled the detection of multiple rodenticides, including warfarin, pindone, chlorophacinone, and bromadiolone, with a total runtime of only 10 s [68].

Overall, the current evidence indicates that no single analytical technique is sufficient to address the complexity of veterinary forensic toxicology. Instead, an integrated, multi-platform approach combining targeted and untargeted methodologies is increasingly necessary to improve detection capabilities, enhance interpretative reliability, and support comprehensive forensic investigations.

## 4. Discussion

**Figure 1:**
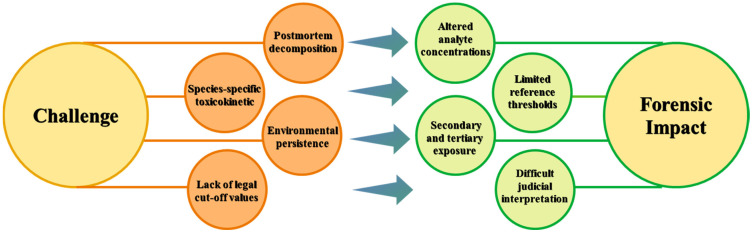
Major interpretative challenges in veterinary forensic toxicology.

Veterinary forensic toxicology has evolved into a critical discipline at the interface of animal welfare, environmental protection, and public health. However, one of the major challenges in the field is the accurate interpretation of toxicological data, which is inherently complex and influenced by multiple factors, including the post-mortem interval, carcass preservation, degree of decomposition, and matrix-dependent analyte stability [3,21,69]. These factors may significantly affect the detectability, concentration, and chemical integrity of toxicants complicating the interpretation of analytical data (Figure 1). Species-specific differences in toxicokinetics, metabolism, and susceptibility, combined with the limited availability of validated reference values and toxicity thresholds, complicate distinction between lethal, sub-lethal, and incidental exposure, particularly in wildlife investigations [14,21].

Consequently, toxicological results should be interpreted within a multidisciplinary forensic framework, integrating analytical data with necropsy findings, histopathological evidence, scene investigation, and relevant circumstantial information to ensure a robust and legally defensible reconstruction of the poisoning event. This holistic approach improves diagnostic accuracy, reduces misinterpretation risk, and strengthens the reliability of veterinary forensic toxicological assessments [1].

The lack of harmonized analytical protocols and interpretative criteria across laboratories remains a major limitation. Recent initiatives aimed at creating national and European networks of veterinary forensic toxicology laboratories have highlighted the importance of shared methodologies and data exchange [16].

The publication of the ANSI/ASB consensus guidelines represents a major step toward the standardization of veterinary post-mortem investigations in cases involving companion animals, livestock, and wildlife [37]. These guidelines define minimum requirements for case intake, chain-of-custody procedures, systematic external and internal examinations, and appropriate sample collection. Early and direct communication between veterinary pathologists and toxicologists is strongly recommended to ensure the collection of suitable specimens in adequate quantities and under appropriate conditions, while the analysis of the documentation of pre-existing diseases, traumatic lesions, and gross pathological findings is essential for the proper contextualization of analytical findings. The adoption of these standardized procedures enhances inter-laboratory consistency, strengthens the evidentiary value of forensic findings in legal contexts, and improves overall the robustness of toxicological interpretation.

Within a One Health approach, veterinary forensic toxicology plays a central role in identifying illegal practices, safeguarding biodiversity, and preventing human exposure to hazardous substances [16,17,20]. By integrating evidence from animal health investigations, environmental monitoring, and public health surveillance systems, this discipline enables the early detection of emerging toxicological threats and supports comprehensive risk assessment at the human–animal–ecosystem interface. Moreover, it provides scientific data to inform regulatory decision-making, guide wildlife conservation strategies, and mitigate environmental contamination, thereby reinforcing its strategic importance in both scientific and policy contexts. Furthermore, this approach will enable the development of innovative analytical strategies that integrate environmental sustainability, promoting greener chemistry while simultaneously enhancing analytical precision, efficacy, and reliability, all in alignment with global health considerations [70].

Building on these advances in standardization, future perspectives in veterinary forensic toxicology are expected to evolve through the enhancement of untargeted substance screening using high-resolution mass spectrometry (HRMS), enabling the detection not only of known xenobiotics but also of emerging contaminants and novel psychoactive substances in biological matrices. It will therefore be essential to systematically implement new toxicokinetic studies across different animal species, with the aim of improving the interpretation and forensic relevance of analytical findings. These developments should be supported by the refinement of standardized guidelines capable of providing widely accepted interpretative frameworks. Achieving these objectives will require coordinated global efforts in scientific collaboration and toxicovigilance.

## 5. Conclusions

Veterinary forensic toxicology has emerged as a pivotal discipline at the interface of animal welfare, environmental protection, and public health. This review highlights the evolution of the field, emphasizing advances in analytical methodologies, the first provision of minimum requirements designed to guide veterinarians performing post-mortem examinations, and the integration of toxicological findings within multidisciplinary forensic frameworks.

A major strength of this study is its focus on recent developments in both companion animal and wildlife toxicology, providing a comprehensive overview of toxicants, analytical strategies, and interpretative challenges. It also addresses emerging contaminants, non-targeted screening using high-resolution mass spectrometry, and the role of One Health approaches in forensic investigations, environmental monitoring, and public health risk assessment. Moreover, this study underscores the importance of harmonized laboratory protocols, early communication between pathologists and toxicologists, and the adoption of international consensus guidelines to enhance the reliability and forensic value of toxicological evidence.

The findings of these studies highlight that intentional animal poisoning remains a significant concern not only for animal health but also for public health. The use of toxic agents can lead to environmental contamination of soil and surface waters and may result in accidental exposure or ingestion, particularly among vulnerable populations such as children, thereby posing substantial risks to human health. By enabling the identification of even low-level exposures, advanced analytical techniques support timely interventions to protect both animal and human health, underscoring the critical role of sensitive and selective methodologies. In this context, the adoption of a One Health approach is essential, as the indiscriminate use of toxic substances represents a complex and far-reaching threat to ecosystems, biodiversity, and human health. Actions that affect the environment without adequate control may compromise the integrity of flora and fauna, ultimately creating indirect yet significant risks for human populations through environmental contamination and exposure pathways.

Overall, this work contributes a timely and integrative perspective that supports evidence-based decision-making in veterinary forensic medicine, informs wildlife conservation strategies, and strengthens global health surveillance. Future perspectives include methodological innovation, expanded toxicokinetic studies across species, and international collaboration that will be essential to further enhance the interpretative robustness, applicability, and societal impact of veterinary forensic toxicology on public and animal health.

In particular, future developments will increasingly focus on the systematic characterization and surveillance of emerging environmental contaminants, including novel pesticides, transformation products, pharmaceuticals, industrial chemicals, and previously unrecognized anthropogenic compounds, which represent a continuously evolving analytical challenge in forensic investigations.

Furthermore, high-resolution mass spectrometry (HRMS)-based untargeted and suspect screening strategies are expected to become central tools in veterinary forensic toxicology, enabling comprehensive chemical profiling, retrospective data mining, and the identification of unknown or unexpected toxicants without prior analytical bias.

The integration of untargeted workflows with expanding spectral libraries, advanced chemometric tools, and machine-learning-assisted data processing will further enhance detection capabilities, supporting more robust interpretation of complex exposure scenarios and improving the sensitivity and specificity of forensic investigations in both biological and environmental matrices.

## Figures and Tables

**Table 1:** Main classes of toxicants involved in veterinary forensic toxicology.

Class	Substances	Affected Species	References
Carbamate pesticides	Carbofuran, Aldicarb, Bendiocarb, Metiocarb	Dogs, cats, birds of prey	[2,3,11,19,26,40,46]
Anticoagulant rodenticides	Brodifacoum, Bromadiolone,	Raptors, carnivores	[3,9,10,11,14,20,21,43,44,45,46,55,56]
Rodenticides	Sodium monofluoroacetate	Predatory wildlife and companion animals	[50]
Molluscicides	Metaldehyde	Dogs	[4,11]
Alkaloids	Strychnine	Companion and wild animals	[11]
Metallic phosphides	Zinc phosphide, Aluminium phosphide	Dogs	[5,39]
Pyrethroids	Bifenthrin, Cypermethrin	Dogs, aquatic fauna	[29,36]
Drugs of abuse	Δ9-tetrahydrocannabinol, amphetamines, synthetic cannabinoids	Dogs, cats	[15,23,24]
Heavy metals	Lead	Avian scavengers	[55,57]
Veterinary pharmaceuticals	NSAIDs, antibiotics, (-)-medetomidine	Raptors, horse	[17,20,28,41]

## Data Availability

No new data were created.
